# Editorial and Miscellaneous

**Published:** 1861-02

**Authors:** 


### EDITORIAL.

### TO THE OFFICERS OF THE AMERICAN MEDICAL ASSOCIATION.

Immediately after the last meeting at New Haven, having noticed that my name was placed upon the Committee on "Prize Essays," I addressed a letter to the President of the Association, informing him that it was uncertain whether I should be able to serve and requesting my name to be withdrawn. As no notice seems, from the volume of transactions, to have been taken of the request, I take this early opportunity of calling your attention to the subject, in order that proper arrangements to prevent mistakes may be made in time.

Respectfully,

D. BRAINARD.

Obituary—Dr. John W. Francis.—In the decease of Dr. John W. Francis—he died at 3 A. M. yesterday, at his residence in East Sixteenth street—New York has lost a marked and shining celebrity. His services to the profession he passionately loved and adorned are at an end; his genial face has faded from mortal view; his eloquence of anecdote, his rare and grand speculations, his noble and hearty benevolence, are no longer living and loved realities. His death was not expected. Though nearly 72 years old, he seemed hale and hearty. Afflicted with carbuncles on the back, he submitted last week to an operation which he thought very much relieved him. The draft on his physical energies proved too great. Suddenly, and without pain, he passed away, retaining his senses to the last.

John Wakeman Francis was born in this city, on the 17th of November, 1789. His father came from Germany, his mother was a Philadelphian, of Swiss descent. At an early age, John was placed in the printing office of George Long, in this city. His ambition soared above type-setting, and he early showed a fondness for books, a love for scholastic pursuits. Employing his leisure time in study, he proved himself an apt as well as vigilant scholar. His genius was recognized, his industry rewarded. Judicious friends came to the aid of the young and striving student, and in 1807 he was enroled to enter the junior class of Columbia College, at the same time commencing the study of medicine under the celebrated Dr. Hosack. In 1809 he graduated, and at once gave his undivided attention to his medical studies. In 1810, in conjunction with Dr. Hosack, he commenced the pnblication of the "American Medical and Philosophical Register," both acting jointly as editors. In 1811 he received his degree of M. D. from the New York College of Physicians and Surgeons—his was among the first degrees granted by this College—and the same year he was admitted to partnership with Dr. Hosack, a partnership which continued through nine years. In 1813 he was appointed to the chair of Materia Medica in his *alma mater*.—*N. Y. Times.*

Dr. Francis occupied successively the chairs of Institutes of Medicine and of Medical Jurisprudence in the College of Physicians in New York.

At the organization of Rutger's Medical College he was appointed Professor of Obstetrics and Forensic Medicine; but this school, notwithstanding the labors of such colleagues as Mott, Hosack and Mitchell, did not succeed, and was closed after four years. His medical writings are numerous and cover a varied field. Perhaps the most successful are his biographical sketches. His edition of Denman's Midwifery was at the time of its publication a valuable addition to the medical literature of this country.

Dr. Francis was a man of genius and honor. His genius linked itself with whatever is great, good or humane. Art, literature, science, found in him an eloquent advocate. But more than his genial social qualities does his sense of honor deserve to be signalized. It is hoped that it may not be deemed as encroaching too much upon his private life to mention that a large part of the proceeds of his labor for many years was devoted to the discharge of obligations in which he became involved through the fault or misfortunes of others, but which he regarded as sacred until they were entirely liquidated.

At the present time, when there are so many to be found who consider it beneath their dignity to pay grocer's and mechanic's bills, but who mourn over the low state of the profession and complain that no respect is shown them by the public, does not this trait deserve especial commendation ?

He was universally respected, beloved by those who had the advantage of his acquaintance of proving him. He honored and never disparaged his noble profession. Dr. Francis was a man of warm religious feelings and convictions, which found genial exercise in the forms of worship of the Episcopal Church, of which he was a member. He was consoled in his last illness by the visits of the Rev. Dr. Hawks, of whose Church he was a member.

---

ETHICS.—One of "ours" wants to know whether it is in accordance with the "code," for prominent members of the American Medical Association to visit patients for *fifty* cents, when the fee bill adopted by their own local society fixes the rate at from $1.50 to $2.00 each. He wants to know whether an *honorarium* of *twenty-five cents*, for prescription and advice at the office, is eminently calculated to "elevate the profession." We must demur to answering under the "Hippocratic oath." The probable reply is, that the fee is *twenty-five* and *fifty cents* to Irishmen and Scandinavians, and the Society fee bill for "the rest of mankind."

We entreat our correspondent to withdraw his harsh expressions, so that we may publish his otherwise exceedingly pertinent and suggestive communication.

---

MATTERS IN DETROIT.—It is a melancholy comment upon the *status* of the profession in our sister city, that the larger proportion of its medical population are applicants for the position of Surgeon to the Marine Hospital in the upper portion of that village, now filled by Prof. Z. Pitcher, of the University of Michigan. Our sympathies are excited especially as we understand that the main chances seem to be divided between Prof. A. B. Palmer, a colleague of Dr. Pitcher, and "Dr." Rudolph, a prominent homœopathic practitioner in the "City of the Straits."

---

Alcohol and Tuberculosis.—Dr. Davis, in the *Transactions*, "invites the critical attention of the Section on Practical Medicine" to the following conclusions:

1st. That the development of tubercular diseases is facilitated by all those agents and influences, whether climatic or hygienic, which directly or indirectly impair or retard the metamorphosis of the organized structures, and the efficiency of the excretory functions.

2d. That observations and carefully devised experiments, both show that the presence of alcohol in the human system, notwithstanding its temporary exhilaration of the cerebral functions, positively retards both metamorphosis and elimination.

3d. That neither the action of alcoholic stimulants on the functions of the human body, nor the actual results of experience, furnish any evidence that these stimulants are capable of either preventing or retarding the development of tubercular phthisis.

These conclusions, being wholly in opposition to generally received opinions on this important subject, certainly demand the attention of practical physicians. Although disposed to disagree with the inferences derived by the learned author from observation of the cases given in support of his present views, we deem the subject one of such general and pervading interest, if not uncertainty, as to warrant the largest latitude of debate and opinion. What is the experience of our correspondents?

---

—The Medical Profession, beyond all others, has remarkably deserved the character of general and extensive knowledge. —*Blackstone*, *Introduction to Commentaries.*

*A Consummation Devoutly to be Wished.*—The New York Academy of Medicine has adopted a resolution discountenancing the publication of its debates and doings in the secular press—a proceeding whereby sundry of the dwarfs were succeeding in attracting public notice by climbing upon the shoulders of some of the real giants of the profession. Let the good work continue.

---

Prof. J. N. McDowell, in the "Transactions of the American Medical Association," observes:

Among the improvements in modern surgery, is Dr. Brainard's mode of causing bones to unite where there has been a want of ossific matter, or an artificial joint formed as a result of a fractured bone.

The mode suggested by Dr. Physick has often, in the hands of the best surgeons proved unsuccessful, viz: Passing a seton through the fracture, but from the experiments of Dr. Brainard on his patients, I am satisfied his is the preferable mode in such cases, although I have not had an opportunity of testing it, and therefore cannot speak from experience. It consists in passing a boring or cutting instrument into the joint, and so exciting the bone as to cause bony matter to be poured out at the point desired. In several cases of the kind, I have been compelled to cut down upon the bone and turn out the ends of the bone and saw them off, an operation which has never failed with me.

I would refer you to his valuable treatise on the subject as published in the "Transactions of the American Medical Association."

Another important improvement has been suggested by Dr. Brainard, and a great number of experiments have been given to prove the position that a poisoned wound can be cured, or the poison prevented from extending by injecting the tincture of iodine around the wound in such a quantity as to neutralize the poison. For accurate information, and the experiments on that subject, I would refer you to his essay, as published. I have not tried it, but believe it to be a good method. At all events, if I were bitten by a rattlesnake or a copperhead, and had the remedies at hand, I would try it and then *get drunk*, if I could; the latter remedy I have never known to fail. It is an Indian remedy, and has been so often tested by them, and I have tested it myself in so many cases, that I believe it to be a safe remedy; but in my own case, I would open the wound, decompose the poison by an alkali applied to the part, try Brainard's remedy, and then *get drunk.*

The application of the alkali, although common, seems scarcely in accordance with sound principles. From the results of Fontana's 6000 experiments, it is evident that alkalies are not antidotal to the venom of serpents. Besides this, it would appear that by their local effect in dissolving the albuminous tissues, they rather tend to promote absorption than to circumscribe the affected part, and thus prevent ready absorption of the virus.

---

*Perspicuity in Medical Writing.*—In looking over the large pile of our exchanges, we have been forcibly impressed by the idea that correspondents do not sufficiently cultivate this truly Christian virtue. We are reminded of the quaint advice of the old writer, which runs somewhat in this wise: If you wish to throw light upon any subject, whether by speaking or writing, it is to be commended that you first gain some smattering thereof yourself; otherwise you will be very like to precipitate yourself into difficulties, from which you will find it quite impossible to extricate yourself *therefrom.*"

---

STIMULANTS.—The Louisville *Journal* beautifully says: "There are times when the pulse lies low in the bosom and beats low in the veins; when the spirit sleeps the sleep, apparently, that knows no waiting in its house of clay, and the window shutters are closed, and the door hung with the invisible crape of melancholy; when we wish the golden sunshine pitchy darkness, and are very willing to fancy 'clouds where no clouds be.' This is a state of sickness when physic may be thrown to the dogs, for we will have none of it. What shall raise the sleeping Lazarus? What shall make the heart beat music again, and the pulses dance to it through all the myriad thronged halls in our house of life? What shall make the sun kiss the Eastern hills again for us, with all its own awaking gladness, and the night overflow with 'moonlight, music, love and flowers?' Love itself is the great stimulant—the most intoxicating of all—and performs all these miracles; but it is a miracle itself, and it is not at the drug store, whatever they say. The counterfeit is in the market, but the winged god is not a money changer, we assure you. Men have tried many things—but still they ask for stimulants. The stimulants we use, but require the use of more. Men try to drown the floating dead of their own souls in the wine cup, but the corpse will rise. We see their faces in the bubbles. The intoxication of drink sets the world whirling again, and the pulses playing wildest music, and the thoughts galloping—but the fast clock runs down sooner; and the unnatural stimulation only leaves the house it fills with wildest revelry, more silent, more sad, more deserted, more dead. There is only one stimulant that never fails, and never intoxicates—Duty. Duty puts a blue sky over every man—up in his heart it may be—into which the skylark, Happiness, always goes singing."

---

*Diphtheria.*—This serious disease, which, from its real presence, (but more especially from the exaggerated rumors put afloat by interested and dishonest parties), has, during the last year or fifteen months, occasioned much popular excitement in this city, now occurs but in rare, sporadic cases. It is no longer to be recognized as, in any sense, epidemic among us. It is gratifying to be able to chronicle the fact that, although a very large proportionate mortality has marked its history under the treatment of the various irregular practitioners—under the judicious measures now generally adopted by the scientific physicians of our city, the *per centage* of deaths has been very moderate—probably not to exceed from *four* to *six* per cent. This result is doubly gratifying to us as journalists, as this journal has, from the inception of the epidemic, steadily sustained those principles of treatment which experience has shown must be relied upon for successful results.

*Berkshire Medical Journal.*—We accidentally omitted to notice in the January number, the advent of a new Medical Journal, of which two numbers have now been received. It is published at Pittsfield, Mass., under the editorial charge of Prof. Wm. Henry Thayer, M. D., and Prof. R. Cresson Stiles, M. D., of the Berkshire Medical College; monthly, at $2.00 *per annum.* We are under especial obligations to this new candidate for professional favor, for the very valuable extract from Prof. Thayer's paper on Diphtheria, which we reproduce in this number. We welcome the *Berkshire Medical Journal* to the list of our exchanges, and heartily trust it will be generously sustained by our Eastern professional brothers.

---

— When Hannibal was engaged in his seige of Rome, the citizens, to show their sense of security against his assaults, and their contempt of his vain efforts, bought and sold the ground upon which his army was encamped, at or even above its previous rates in the market. So it is now with regard to the little grounds of truth upon which the many false systems of medical doctrine, which attempt the hopeless task of overturning legitimate medicine, are encamped. We shall not abandon those truths, but hold them in equally as high estimation, as though they had never been defiled or dishonored by the footstep or grasp of any short-lived delusion, or evanescent cheat, clad in the attire of medicine.

---

*The Balance of Account.*—The balance of account between satisfaction and remorse, was jocosely stated by Dr. Warren to Lady Spencer, who had said she thought the frequent reflection that a different treatment might have saved their patients, must embitter the lives of medical men, he told her that the balance was greatly in favor of satisfaction, for he hoped to cure her *forty* times before he killed her *once.—Physic and Physicians.*

*Shaftesbury Paraphrased.*—Many succeed in *pulling down* to a miracle, who have miserably fallen in attempt to build up. Though compassion in real war may make the ruinous practice less delightful, 'tis certain that in the medical warring world, the springing of mines, the blowing up of towers, of bastions and ramparts of Medicine, with *systems*, hypotheses, *opinions* and *doctrines* into the air, is a spectacle of all others the most naturally rejoicing.

---

— The *American Medical Association*, although in danger of being robbed of some of its *prestige* as a National Assemblage, by the progress of the secession movement, seems quite likely notwithstanding to prove of some ultimate use. If it has not accomplished all at first proposed, it has at all events profited by learning what to avoid in its future course. Wrangling over the manifold details of the code—ventilation of conflicting notions as to the proper manner of conducting medical colleges—petty squabbles between sectional interests—all that sort of thing will probably hereafter be wholly ignored. The Association will lend its energies to the real work of developing the Science and Art of Medicine. Unfortunately, notoriety is more easily acquired by "rising to points of order," or by indulging in acrimonious personalities of debate, than by the slowly accumulating results of industrious observation and study. We "indulge a trembling hope," that hereafter the National convocation may cultivate the latter to the neglect of the former.

---

EXPERIENCE.—Experience has its principles as well as philosophy; for experience *is* philosophy—the philosophy of common sense; and it is well that you should know that these principles are nothing more than the aphorisms, proverbs, maxims, and wise sayings, current in the world. These are the pith and essence of multitudinous experiments, experience, professional, moral, domestic, or national, as well as physiological and pathological. In a word, they constitute empirical knowledge. The proverb, to mention a solitary instance, of "Conceit (or the fancy, imagination) will kill, and conceit will cure," is an embodiment of the philosophy of globulistic and mesmeric therapeutics. The acquisition of a certain amount of medical knowledge, by uninstructed daily experience, is equally illustrated by the proverbial saying, "A man is either a fool or a physician at forty."

What, then, is the requisite conduct for the attainment of the wise experience of which I speak? Simply this: long-continued, sedulous, accurate observations of manifest external phenomena—observation independently of aids, and therefore prompt, because practicable under all circumstances, in which the eyes, the ears, and senses generally can co-operate with the instinctive exercise of the judgment, or common sense, as it is termed. There must be the practised eye and ear, trained by long use; and practised judgment, trained by careful exercise. All men have not the natural qualities; many want the industry; but to those who have both, experience will afford a power of intuition such as is is sometimes really marvellous in its results.—*Laycock on Medical Observation.*

---

*Transactions of the American Medical Association*, Vol. 13, 1860, pp. 930. Printed for the Association.

The current volume of the Transactions is beautifully printed, with clear type, upon good paper. Besides the usual formal records of the annual session, it contains:

Report on the Medical Topography and Epidemics of New York, by Joseph H. Smith, M. D.; Report on *ditto* of North Carolina, by James H. Dickson, M. D.; On various Surgical Operations, for relief of defective vision, by M. A. Pallen, M. D., of St. Louis; On the Improvements in Surgery during the last Fifty Years, by Joseph N. McDowell, M. D., of St. Louis; On Morbus Coxarius, by L. A. Sayre, M. D.; On the Influence of Alcoholic Drinks on the Development of Pulmonary Tuberculosis, by N. S. Davis, M. D.; Education of Imbecile and Idiotic Children, by H. P. Ayres, M. D., of Indiana; On Inebriate Asylums, by C. McDermont, M. D., of Ohio; Medical Education, by D. M. Reese, M. D. etc., New York; Medical

Literature, by D. F. Wright, M. D., Tennessee; On Medical Necrology, by Christopher C. Cox, M. D., Maryland; Plan of Organization; Code of Ethics, etc., etc.

Many of these papers exhibit much industry in preparation, and are substantially valuable. We shall find occasion for inserting some extracts hereafter.

---

THE YEAR BOOK OF AMERICAN CONTRIBUTIONS TO MEDICAL SCIENCE AND LITERATURE.

Dr. O. C. Gibbs wishes us to say that, because of the money crisis and the unsettled state of National affairs, his proposed *Year Book* will be delayed for a time. He is, however, in hopes to issue sometime during the coming three months. So soon as *one thousand* names (1000) are received as subscribers, the work will be put to press. Thankful for the kind words spoken for him by nearly all journals, and the expression of encouragement given by subscribers, he must still ask his friends to continue to labor in his behalf. He has spent much time and money in starting an enterprise that he had hoped would meet a professional want, and do honor to American Medicine. A professional expression of sympathy and encouragement is all that is wanted to insure success and perpetuity to the enterprise. Every physician in the country should become a subscriber; but if one in ten will aid the enterprise, a *summary* of American Medical Literature and progress will be given to the profession. We know of no one work, of ten times the cost, that will give the same amount of information. Terms $3.00 per year, to be paid on the issue of the work. Subscribers should direct at once to O. C. Gibbs, M. D., Frewsburg, N. Y.—*Com.*

---

— To which does Burton refer, physicians or patients, when he writes: *Qui medice vivit, misere vivit?*

### OBITUARY.

At a meeting of the students of Rush Medical College, held in the Lecture Room, Feb. 12th, for the purpose of passing resolutions expressive of their sympathy with Prof. Miller on the death of Mrs. Miller, on motion B. Ward was elected President, and M. Reece, Secretary. A Committee on Resolutions was appointed, consisting of Messrs. Clark, Russell, Buck and Comstock, who reported the following resolutions, which were unanimously adopted:

Whereas, It has been announced to us that our friend and instructor, Prof. De Laskie Miller, has been deeply afflicted in the loss of his companion, and

Whereas, It has been our pleasure to know and appreciate her high social and intellectual qualities, therefore,

*Resolved*, That we tender him our sympthy and condolence in this great bereavement, and although we cannot know the plenitude of sorrow which fills *his* heart, yet we feel that society has lost one of its brightest ornaments, a true woman, and one whose name is embalmed in the memory of every member of this class. While we commingle our tears with those of the afflicted family, we commend them for strength and consolation to that Power who is Love, and who doeth all things well.

*Resolved*, That, if agreeable to the wish of our bereaved friend, we will attend the funeral of the deceased as a class.

*Resolved*, That a copy of these resolutions be transmitted by the President and Secretary to the Prof. Miller, and also to the *Chicago Medical Journal*, and daily papers, for publication.

B. WARD, President.

M. Reece, Secretary.

E. A. Clark,<br>E. F. Russel,<br>S. S. Buck,<br>J. D. Comstock, } Committee.

*Aphorism for Medical Students.*—That which is once clearly understood can scarcely be forgotten; whereas that which is not understood or misunderstood, it were well it should be banlshed from the memory as speedily as possible.

— "The poets had a clear idea of the folly of preferring the mountebank and witch to the physician, when they made *Æsculapius* and *Circe*, brother and sister, both children of the Sun, as in the verses—

*Ipse repertorem medicinæ talis et artis*
*Fulmine Phœbigenam stygia detrusit ad undas.*
—Æneid, VII, 772.

And again—

*Dives inacccssos ubi Solio filia lucos*, &c.—Ibid., 11."
—*Bacon Adv. Learning*, *B II.*

*To Business Men.*—Your attention is requested to this Journal as an advantageous advertising medium. Enjoying as it does, the largest circulation of any professional journal in the North-West, and with constant additions to its subscription list—going into almost every town and city of any importance in this section of the country—a notice in its advertising columns will be sure of vastly greater publicity than usually befalls advertisements in most periodicals. Terms as moderate as could reasonably be desired.

| | | | |
|---|---|---|---|
| Half page, one insertion, | $5 00 | Six months, $20 00 | One year, $30 00 |
| Whole page, " " | $8 00 | " " $25 00 | " " $40 00 |

Communications.—All communications for insertion in the Journal, or on business matters connected therewith, may hereafter be directed to the Junior Editor,

DR. J. ADAMS ALLEN,
Box 4458, Chicago, Illinois.

